# Provincial variability in congenital heart disease prevalence in Argentina, 2014–2019: A population-based analysis from national registry data

**DOI:** 10.1371/journal.pgph.0005217

**Published:** 2026-06-22

**Authors:** María Clara Vita, Nicolás Fantozzi, Federico Méndez Ortiz, Ludmila Di Lullo, Romina Kokal, Simbad Peralta, Sandra Di Lalla

**Affiliations:** Hospital General de Niños Pedro de Elizalde, Buenos Aires, Argentina; Washington State University, UNITED STATES OF AMERICA

## Abstract

Congenital heart defects are a leading cause of neonatal morbidity and mortality worldwide. Early diagnosis is essential to enable timely treatment and improve patient outcomes; however, access to early detection and specialized care is unevenly distributed across regions in Argentina. This study describes the prevalence and timing of diagnosis of congenital heart defects and critical congenital heart defects among live births covered by the public health system nationwide. We performed a cross-sectional, descriptive study based on province-level aggregated data. The analysis included all live births between 2014 and 2019 with a diagnosis of congenital heart defects reported up to five years of age in the National Registry of Congenital Heart Diseases. We calculated prevalence rates and median age at case notification for each province to assess geographic disparities. Out of 2,473,720 live births, 16,150 cases of congenital heart defects (prevalence 65.3 per 10,000 live births) and 3,700 cases of critical congenital heart defects (15.0 per 10,000) were identified. Provincial prevalence ranged widely, from 34.9 to 212.2 per 10,000 for congenital heart defects and from 10.7 to 27.3 per 10,000 for critical cases. The national median age at notification was 75 days for all congenital heart defects and 29 days for critical cases, with notable provincial differences. These findings demonstrate significant provincial variability in both the prevalence of congenital heart defects and the age at which cases are reported to the national health registry. Strengthening early detection efforts and ensuring equitable access to specialized care are crucial to reduce morbidity and mortality associated with these conditions in Argentina and similar settings.

## Introduction

Congenital heart defects (CHD) are structural or functional abnormalities of the heart that originate during prenatal development and can be detected before or after birth. They represent the most common congenital malformation in newborns and are a leading cause of infant mortality and disability worldwide [1]. In Argentina, congenital heart defects are the second leading cause of death in children under one year of age, accounting for over 10% of deaths in this age group [2].

Early diagnosis is possible through neonatal screening using pulse oximetry and prenatal diagnosis, which can identify most congenital heart defects from the 20th week of gestation [3]. Advances in cardiology, cardiovascular surgery, and postoperative care have improved survival and quality of life for children with complex heart defects.

Approximately one in 500 live births is affected by critical congenital heart defects (CCHD), defined as ductus-dependent anomalies that can cause death or require invasive procedures within the first 28 days of life [4,5]. These conditions pose significant clinical and public health challenges, especially in low- and middle-income countries where access to specialized care is limited and mortality rates remain high [6].

Given that nearly half of children with congenital heart defects require surgery within their first year of life and that timely diagnosis and treatment could benefit up to two-thirds of these patients, ensuring equitable access to care is essential. Barriers include limited infrastructure and human resources within the public health system to meet the national demand for diagnosis and treatment [7].

To address these gaps, Argentina created the National Congenital Heart Disease Program in 2008, establishing a coordinated referral network with designated provincial diagnostic centers and a smaller number of high-complexity surgical centers [7,8]. During the study period, the network comprised 40 diagnostic centers distributed across all 23 provinces and the Autonomous City of Buenos Aires (CABA). In contrast, the 15 surgical centers were concentrated in only a limited number of locations nationwide (eight provinces and CABA), creating heterogeneous catchment areas and potentially influencing detection and treatment patterns. Within this context, this study analyzes the performance of the national network by estimating the prevalence of CHD and CCHD among live births in the public health sector from 2014 to 2019, and by examining differences in their distribution across Argentine provinces.

## Materials and methods

### Study design and population

We conducted a cross-sectional, descriptive study based on province-level aggregated data, including all 23 provinces and CABA. The dataset included all live births diagnosed with congenital heart defects and registered in the National Registry of Congenital Heart Diseases (RNCC), part of the Argentine Integrated Health Information System (SISA), between January 1, 2014, and December 31, 2019. Only cases reported within the first five years of life were included. Individual case records were used exclusively to construct provincial indicators; no individual-level analyses were performed. Patients of non-Argentine nationality diagnosed within the national territory were excluded.

### Definition of critical congenital heart defects

Critical congenital heart defects (CCHD) were defined as ductus-dependent structural cardiac anomalies requiring surgical or invasive intervention (surgery or cardiac catheterization) within the first 28 days of life to prevent death. Without timely intervention, mortality and survival with significant disability are extremely high [4,9]. The critical defects included in this study were: aortic arch hypoplasia, aortic valve atresia, coarctation of the aorta, coronary anomaly (including ALCAPA – Anomalous Left Coronary Artery from the Pulmonary Artery), Ebstein’s anomaly, hypoplastic left heart syndrome, interruption of aortic arch, pulmonary atresia, single ventricle, transposition of the great arteries, tetralogy of Fallot, total anomalous pulmonary venous return, and truncus arteriosus.

### Data sources

- National Registry of Congenital Heart Diseases (RNCC) — SISA module [10]
- National Live Births Registry from the Ministry of Health — Directorate of Health Statistics and Information (DEIS) [11]
- Health workforce data from Argentine Ministry of Health, based on publicly available datasets on certified health professionals by specialty and province [12]

### Variables

- Cumulative prevalence of CHD: calculated as the total number of registered cases during 2014–2019 divided by the total number of live births occurring in public health facilities during the same period.
- Cumulative prevalence of CCHD: calculated similarly but considering only cases classified as critical. Both prevalences are expressed per 10,000 live births and stratified by province and Buenos Aires City.
- Age at diagnosis: extracted from the RNCC based on the date of case notification. This date may not always correspond exactly to the clinical detection date; therefore, delays in notification could introduce bias in estimated age at diagnosis.
- Sociodemographic variables: province of usual residence of the patient was included to analyze regional prevalence distribution.

### Procedure

Registered variables included diagnosis or notification date, age at notification, type of CHD, and province of residence. Prevalence estimates at the national and provincial levels were calculated using the total number of cases registered relative to live births in official health establishments according to DEIS data for 2014–2019. Prevalences were calculated assuming a Poisson distribution and expressed per 10,000 live births with 95% confidence intervals.

### Methodological considerations

- Data quality control: RNCC records were validated based on completeness and internal consistency criteria. Duplicate records were excluded from analysis.
- Limitations: Prevalence estimates may be affected by underreporting in some provinces, as reporting to the RNCC depends on the timeliness of data entry by each health facility. Although the system registers both the date of birth and the date of case notification, delays between detection and reporting are expected and may affect the variable *“age at diagnosis,”* since cases may be entered later than the actual moment of identification. The RNCC primarily includes notifications from public sector facilities, as reporting from private institutions was not mandatory nationwide during the study period; therefore, results mainly reflect the public subsector. Public sector live births were used as the denominator to improve accuracy of estimates.

### Ethical considerations

This study was conducted using secondary data from the RNCC of SISA. Data were accessed for research purposes between 1 March 2022 and 30 June 2022. Although the original dataset contained administrative variables that could potentially identify individual participants, the authors did not use or retain any such information. All analyses were conducted on de-identified data, ensuring confidentiality. The study protocol was reviewed and approved by the Ethics Committee of the Hospital General de Niños Pedro de Elizalde in Buenos Aires, in accordance with the principles of the Declaration of Helsinki (approval number: 6627).

## Results

**Fig 1:**
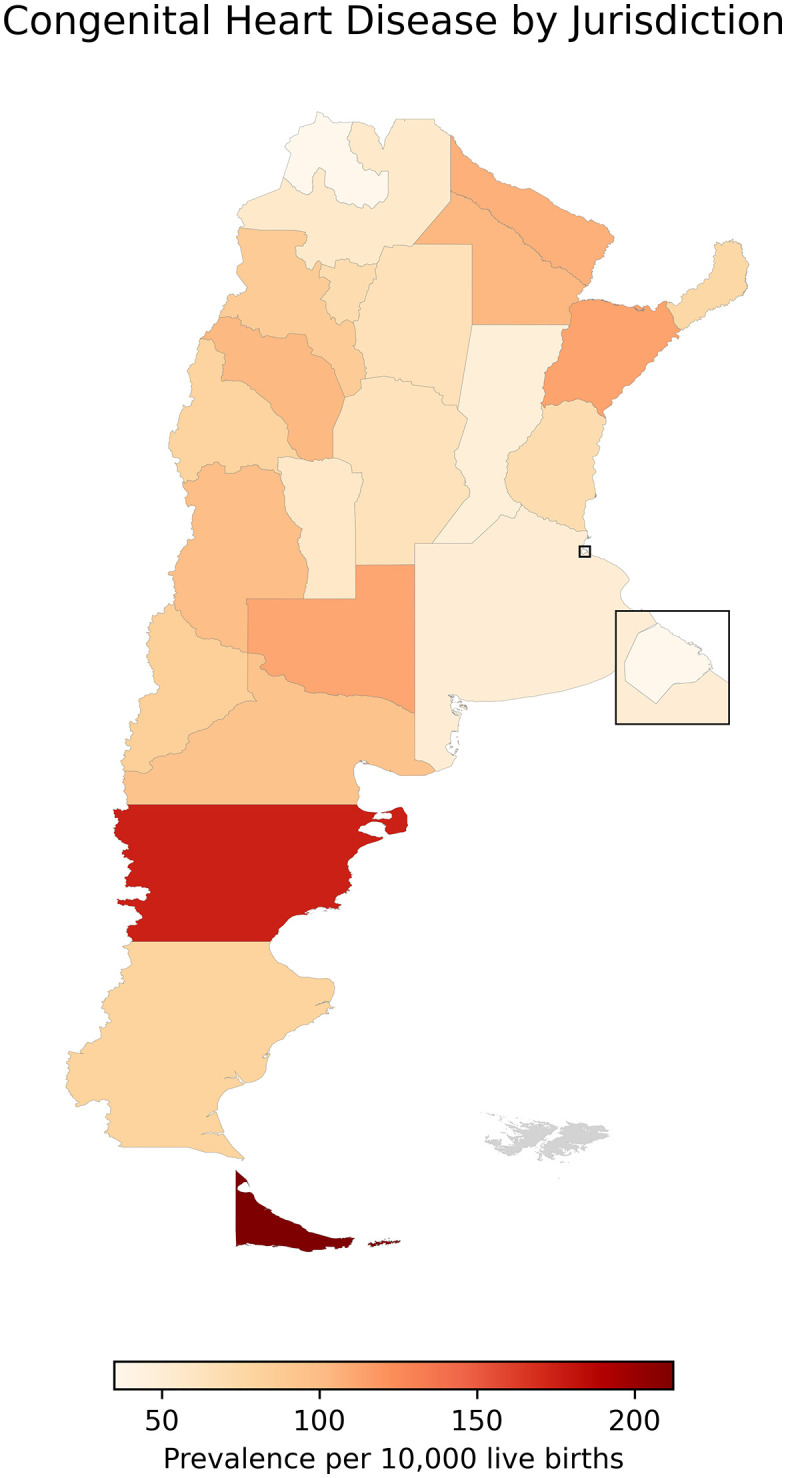
Spatial distribution of congenital heart disease (CHD) prevalence across Argentine provinces (2014–2019). Basemap source: GeoRef API – Secretaría de Innovación, Ciencia y Tecnología (Jefatura de Gabinete de Ministros), https://apis.datos.gob.ar/georef/api/v2.0/provincias.geojson; CC BY 4.0.

Between January 1, 2014, and December 31, 2019, a total of 2,473,720 live births were recorded in public health facilities, with 16,150 cases of CHD identified. The cumulative prevalence was 65.3 per 10,000 live births (95% CI: 64.3–66.3). The lowest provincial prevalences were observed in Jujuy (34.9; 95% CI: 30.1–40.4), Buenos Aires City (CABA) (35.2; 95% CI: 31.3–39.7), and Santa Fe (47.7; 95% CI: 44.4–51.2), while the highest prevalences were recorded in Corrientes (112.6; 95% CI: 105.6–120.1), Chubut (174.9; 95% CI: 158.3–193.1), and Tierra del Fuego (212.2; 95% CI: 183.0–246.1) (Table 1). The spatial distribution of these provincial prevalences is shown in Fig 1.

**Table 1:** Prevalence of congenital heart disease (CHD) and critical congenital heart disease (CCHD) by province. Argentina, 2014–2019.

Province	No. of CHD cases	No. of CCHD cases	CCHD as % of CHD	Official Sector Births	CHD prevalence (per 10,000) (95% CI)	CCHD prevalence (per 10,000) (95% CI)
**Jujuy**	176	68	38.6	50,451	**34.9** (30.1 - 40.4)	**13.5** (10.3 - 16.9)
**Santa Fe**	755	249	33.0	158,388	**47.7** (44.4 - 51.2)	**15.7** (13.8 - 17.7)
**CABA**	268	88	32.8	76,030	**35.2** (31.3 - 39.7)	**11.6** (9.2 - 14.0)
**Tucumán**	716	217	30.3	101,934	**70.2** (65.3 - 75.6)	**21.3** (18.5 - 24.1)
**Santiago del Estero**	513	144	28.1	78,724	**65.2** (59.8 - 71.0)	**18.3** (15.4 - 21.3)
**Córdoba**	1008	279	27.7	157,310	**64.1** (60.3 - 68.1)	**17.7** (15.7 - 19.8)
**San Luis**	137	38	27.7	23,984	**57.1** (48.3 - 67.5)	**15.8** (10.8 - 20.9)
**Buenos Aires**	4824	1293	26.8	971,993	**49.6** (48.3 - 51.1)	**13.3** (12.6 - 14.1)
**San Juan**	379	98	25.9	47,843	**79.2** (71.7 - 87.6)	**20.5** (16.4 - 24.5)
**Entre Ríos**	492	114	23.2	70,668	**69.6** (63.8 - 76.0)	**16.1** (13.3 - 19.1)
**Misiones**	708	161	22.7	95,170	**74.4** (69.1 - 80.1)	**16.9** (14.4 - 19.5)
**Salta**	641	136	21.2	113,945	**56.3** (52.1 - 60.8)	**11.9** (10.0 - 14.0)
**Mendoza**	1029	216	21.0	103,795	**99.1** (93.3 - 105.4)	**20.8** (18.1 - 23.6)
**Catamarca**	196	40	20.4	22,574	**86.8** (74.5 - 99.8)	**17.7** (12.2 - 23.2)
**La Rioja**	209	40	19.1	20,442	**102.2** (89.3 - 117.0)	**19.6** (13.5 - 25.6)
**La Pampa**	175	29	16.6	15,710	**111.4** (96.1 - 129.0)	**18.5** (11.7 - 25.2)
**Neuquén**	297	45	15.2	35,345	**84.0** (75.0 - 94.1)	**12.7** (9.1 - 16.6)
**Chaco**	989	134	13.5	96,172	**102.8** (96.7 - 109.4)	**13.9** (11.6 - 16.3)
**Santa Cruz**	216	29	13.4	27,124	**79.6** (69.7 - 90.9)	**10.7** (6.8 - 14.6)
**Tierra del Fuego**	171	22	12.9	8,057	**212.2** (183.0 - 246.1)	**27.3** (15.9 - 38.7)
**Formosa**	539	67	12.4	50,637	**106.4** (97.9 - 115.8)	**13.2** (10.1 - 16.4)
**Chubut**	381	45	11.8	21,789	**174.9** (158.3 - 193.1)	**20.7** (14.7 - 27.1)
**Río Negro**	420	48	11.4	44,735	**93.9** (85.4 - 103.3)	**10.7** (7.8 - 13.9)
**Corrientes**	911	100	11.0	80,900	**112.6** (105.6 - 120.1)	**12.4** (10.0 - 14.8)
**Total**	**16,150**	**3,700**	**22.9**	**2,473,720**	**65.3 (64.3 - 66.3)**	**15.0 (14.5 - 15.4)**

**Notes:** CHD: congenital heart disease. CCHD: critical congenital heart disease. Prevalences are expressed per 10,000 live births in the public sector, 2014–2019. CI: 95% confidence interval.

The national median age at the time of CHD case notification was 75 days (interquartile range [Q1–Q3]: 21–214). Provinces with the earliest notifications were San Juan (31 days; Q1–Q3: 6–127), Córdoba (39 days; Q1–Q3: 11–204), and Formosa (42 days; Q1–Q3: 9–120), whereas the highest median ages were found in Jujuy (101 days; Q1–Q3: 20–330), Santiago del Estero (121 days; Q1–Q3: 42–291), and La Rioja (147 days; Q1–Q3: 48–326) (Table 2).

**Table 2:** Median age at notification of congenital heart disease (CHD) and critical CHD (CCHD), and median age ratio (CCHD/CHD), by province, Argentina, 2014–2019.

Province	CHD – Median age (days, Q1–Q3)	CCHD – Median age (days, Q1–Q3)	Age ratio (CCHD/CHD)
**La Pampa**	**94** (34 - 240)	**7** (1 - 154)	0.07
**La Rioja**	**147** (48 - 326)	**14** (3 - 142)	0.10
**Misiones**	**73** (21 - 220)	**12** (3 - 103)	0.17
**Chubut**	**78** (35 - 154)	**15** (6 -75)	0.19
**Jujuy**	**101** (20 - 330)	**23** (3 - 119)	0.22
**San Luis**	**63** (16 - 208)	**14** (7 - 45)	0.22
**Santa Cruz**	**59** (21 - 128)	**13** (2 - 35)	0.22
**Neuquén**	**60** (17 - 172)	**14** (3 - 69)	0.23
**Santa Fe**	**79** (18 - 276)	**18** (5 - 140)	0.23
**Tucumán**	**77** (20 - 184)	**19** (5 - 107)	0.25
**Corrientes**	**75** (36 - 157)	**22** (5 - 138)	0.29
**Salta**	**73** (21 - 198)	**23** (5 - 112)	0.31
**San Juan**	**31** (6 - 127)	**10** (2 - 73)	0.31
**Entre Ríos**	**80** (21 - 240)	**27** (5 - 137)	0.34
**Mendoza**	**52** (16 - 213)	**18** (4 - 100)	0.35
**Río Negro**	**92** (33 - 214)	**36** (7 - 157)	0.39
**Buenos Aires**	**93** (26 - 258)	**39** (7 - 181)	0.42
**CABA**	**100** (30 - 249)	**46** (11 - 217)	0.46
**Catamarca**	**93** (43 - 219)	**46** (14 - 125)	0.50
**Chaco**	**54** (20 - 175)	**29** (5 - 206)	0.54
**Córdoba**	**39** (11 - 204)	**25** (4 - 190)	0.64
**Tierra del Fuego**	**76** (25 - 180)	**54** (8 - 126)	0.71
**Santiago del Estero**	**121** (42 - 291)	**106** (50 - 236)	0.87
**Formosa**	**42** (9 - 120)	**59** (9 - 223)	1.40
**Total**	**75 (21 - 214)**	**29 (6 - 159)**	**0.39**

**Notes:** CHD: congenital heart disease. CCHD: critical congenital heart disease. Age at notification: days of life. Q1–Q3: first and third quartiles. Age ratio: values <1 indicate earlier notification of CCHD compared with CHD; values ≥1 indicate similar or later notification.

During the study period, 3,700 cases of CCHD were registered, yielding a prevalence of 15.0 per 10,000 live births (95% CI: 14.5–15.4). The lowest provincial prevalences were recorded in Río Negro (10.7; 95% CI: 7.8–13.9), Santa Cruz (10.7; 95% CI: 6.8–14.6), and CABA (11.6; 95% CI: 9.2–14.0), while the highest prevalences were observed in Mendoza (20.8; 95% CI: 18.1–23.6), Tucumán (21.3; 95% CI: 18.5–24.1), and Tierra del Fuego (27.3; 95% CI: 15.9–38.7) (Table 1).

The national median age at CCHD case notification was 29 days (Q1–Q3: 6–159). Provinces with the earliest median notification ages were La Pampa (7 days; Q1–Q3: 1–154), San Juan (10 days; Q1–Q3: 2–73), and Misiones (12 days; Q1–Q3: 3–103), while the highest median notification ages were recorded in Tierra del Fuego (54 days; Q1–Q3: 8–126), Formosa (59 days; Q1–Q3: 9–223), and Santiago del Estero (106 days; Q1–Q3: 50–236) (Table 2).

The median age ratio (CCHD/CHD) varied substantially across provinces, ranging from 0.07 to 1.40 (Table 2). Values below 1 indicate earlier notification of CCHD relative to CHD. In most provinces, median age at notification was lower for CCHD than for CHD, although the magnitude of this difference varied widely. The highest value was observed in Formosa (1.40), indicating later notification of CCHD compared with CHD, while values were also close to 1 in Santiago del Estero (0.87), suggesting limited temporal advantage in the notification of critical cases.

## Discussion

This study highlights substantial provincial heterogeneity across Argentina in CHD and CCHD prevalence, the proportion of critical cases among total CHD cases, and median age at notification. By moving beyond national estimates, it examines how these indicators vary across provinces, showing how aggregate figures can obscure important territorial differences in case ascertainment. Unlike broader congenital anomaly surveillance systems, this analysis draws on a national registry specifically dedicated to CHD. Taken together, these metrics offer a systems-level perspective on how differences in detection, reporting, and access to care may shape observed patterns across provinces. In this context, median age at notification provides an additional indicator on the timeliness with which cases enter diagnostic and surveillance pathways.

Compared with prior Argentine and international studies, the overall CHD prevalence observed in our study was 65.3 per 10,000 live births, lower than the 81.7 per 10,000 reported in Europe [13]. In contrast, the CCHD prevalence was 15.0 per 10,000 live births, higher than the 11.4 per 10,000 reported by Groisman et al. using data from Argentina’s National Network of Congenital Anomalies (RENAC) [14], and the 10.1 per 10,000 reported by Bakker et al. in an international analysis [15]. These differences likely reflect not only epidemiologic variation, but also differences in surveillance design, inclusion criteria, and case ascertainment across settings. In particular, registries differ in population coverage, data sources, and the time window during which cases can be identified, all of which may substantially affect measured prevalence. European estimates also include stillbirths and pregnancy terminations due to fetal anomalies [13], whereas our study period (2014–2019) predated the promulgation of Argentina’s Voluntary Termination of Pregnancy Law (Law 27.610, December 2020). These findings should therefore be interpreted within that pre-law context. In Latin America, congenital anomalies account for an increasing share of infant mortality as countries undergo epidemiological transition, within health systems that are often mixed and segmented across public, social security, and private sectors [16]. Surveillance systems for congenital anomalies also vary substantially across the region in coverage, case ascertainment, and reporting structure, all of which can influence measured prevalence [17,18]. Argentina shares several of these features, but the National Congenital Heart Disease Program established a coordinated nationwide referral and surveillance structure for CHD that allows case notification beyond the perinatal period, broadening ascertainment compared with hospital-based surveillance systems limited to anomalies detected during the maternity stay.

The marked provincial variation observed in CHD and CCHD prevalence is unlikely to reflect only true epidemiologic differences. Detection of CHD depends on several structural factors, including access to prenatal diagnosis, postnatal clinical suspicion, confirmatory echocardiography, and the capacity of local services to identify defects across the full spectrum of severity. This is particularly relevant for non-critical CHD, which is more easily under-detected when diagnostic infrastructure is limited or when less symptomatic lesions do not reach specialist evaluation. In this context, the proportion of CCHD among all CHD cases becomes analytically informative. According to the literature, CCHD account for approximately 25% of all CHD [9]; in our study, the national proportion was 23%, with substantial heterogeneity across provinces (Table 1). A relatively high critical-case proportion may indicate preferential detection of severe lesions together with under-ascertainment of milder or non-critical defects, whereas unusually low proportions may suggest missed critical cases, incomplete capture of severe disease, limited access to specialized diagnostic services, or early neonatal death before diagnosis or notification. In this sense, the proportion of CCHD relative to total CHD may provide an additional indicator of case ascertainment quality, with values closer to the expected 25% suggesting more balanced detection across severity levels. Other contextual factors, including differences in access to services, socioeconomic conditions, and environmental or genetic factors, may also contribute, but these could not be examined with the available data [19].

Median age at notification captures the time from birth to case notification in the surveillance system, reflecting how early a CHD case enters the health system’s detection and reporting pathway. In Argentina, where SISA allows case notification beyond the perinatal period rather than restricting it to the maternity stay, this indicator provides a broader view of when cases are effectively detected and recorded. Although notification age does not necessarily coincide with the exact clinical moment of diagnosis, it provides a useful approximation of the timeliness of detection, confirmation, and registration at the provincial level. This is particularly relevant for CCHD, for which the national median age at notification was shorter than for CHD overall (29 vs 75 days). This pattern is consistent not only with the greater clinical severity and detectability of critical defects, but also with the organization of the National Congenital Heart Disease Program, which was designed to facilitate identification and referral of cases requiring timely surgical intervention. The median age ratio (CCHD/CHD) captures the relative timing of notification between critical and non-critical cases across provinces. Even so, delays in CCHD notification remain clinically meaningful, as timely recognition in the first weeks of life is critical and may influence early survival, although this study did not directly assess mortality outcomes [4,6]. In settings with delayed or limited access to specialized care, some newborns with CCHD may not be diagnosed or may not survive long enough to be captured by surveillance systems, potentially contributing to underestimation and shaping observed notification patterns. In this context, systematically later notification may reflect gaps in early detection pathways, including prenatal diagnosis, postnatal clinical recognition, pulse oximetry screening, access to confirmatory testing, or timely referral, consistent with evidence that a substantial proportion of CHD cases are diagnosed beyond the neonatal period and even into adulthood [20]. Earlier notification, in contrast, may indicate more effective implementation of these processes. The relationship between policy implementation and measurable outcomes can be illustrated by neonatal pulse oximetry screening, an established strategy for early detection of CCHD [5]. In our data, La Pampa, which implemented mandatory screening in 2017, showed the earliest median age at notification for CCHD (7 days), while CABA (46 days) and Entre Ríos (25 days) showed later notification despite similar legal frameworks. These disparities underscore that while enacting laws is a necessary step, rigorous implementation and continuous monitoring are required to translate policy into timely clinical action. This example reinforces the utility of median age at notification as a performance benchmark: it not only helps identify where screening strategies may be functioning more effectively, but also highlights where structural barriers may be delaying the transition from detection to registry notification.

These differences in notification timing may reflect underlying structural differences in healthcare capacity across provinces. In particular, variation in the availability of specialized human resources and in the organization of referral networks likely play a central role in shaping both detection and reporting of CHD and CCHD. Available administrative data from the Argentine Ministry of Health suggest that pediatric cardiology specialists are concentrated in a limited number of urban centers, particularly in Buenos Aires and CABA, with substantially lower availability in several provinces [12]. This uneven distribution may influence where and how CHD is detected, as timely diagnosis in low-resource settings may depend on referral to higher-complexity centers rather than local specialist evaluation. In these contexts, fragmented pathways for referral, diagnostic confirmation, and registration may contribute to later notification and under-ascertainment, especially for defects that are less symptomatic at presentation. Thus, part of the observed heterogeneity may reflect not only differences in early detection, but also differences in access to specialized pediatric cardiology and in the functioning of referral systems. Although the National Congenital Heart Disease Program provides a structured referral network, its effectiveness still depends on the uneven availability of local diagnostic capacity across provinces.

The observed patterns should be interpreted in light of the structure of the surveillance system. During the study period, notification within SISA was implemented primarily through public-sector providers, while reporting from the private sector was not uniformly mandatory, which may have resulted in some under-ascertainment in jurisdictions with a larger private-sector share, such as CABA. In many Argentine provinces, a considerable share of live births occurs in public facilities (according to national vital statistics from DEIS), supporting the use of public-sector births as a robust denominator for provincial prevalence estimates. Accordingly, any upward bias introduced by cross-subsector notification is expected to be limited and unlikely to fully explain the observed between-province variability. In addition, because the registry is embedded within a referral network for CHD care, defects requiring timely intervention may have been captured more consistently than milder conditions, potentially influencing both prevalence estimates and the proportion of CCHD among total CHD. Prevalence estimates were based on live births registered in Argentina, excluding children born abroad who were later diagnosed in the country. Including foreign-born cases could lead to overestimation of prevalence relative to the national birth cohort; restricting the denominator to births registered in Argentina improves alignment between cases and population at risk.

Beyond their descriptive value, the three metrics analyzed in this study, overall CHD prevalence, the proportion of CCHD among total CHD, and median age at notification, can function as complementary operational indicators of health-system performance. Lower-than-expected prevalence may indicate incomplete case ascertainment, while unusually high or low proportions of CCHD may reflect imbalances in the detection of severe versus milder defects. Median age at notification adds a temporal dimension, capturing how early cases enter diagnostic and reporting pathways and helping to identify delays in screening, confirmatory evaluation, or referral. Considered together, these indicators help distinguish provinces where under-ascertainment predominates from those where delays in detection or selective capture of more severe cases are more prominent. This is clinically relevant because earlier detection enables timely stabilization and referral, while prenatal identification can improve perinatal planning for high-risk newborns [21,22]. Routine analysis of registry-based metrics can support monitoring of referral networks, screening implementation, and diagnostic capacity across provinces, and may also be relevant for other middle-income settings with similarly fragmented health systems.

These findings suggest that provincial heterogeneity in CHD and CCHD reflects not only epidemiological variation, but also differences in how cases are detected and reported. Considering prevalence together with severity distribution and notification timing helps differentiate between under-ascertainment, delayed detection, and selective capture of severe disease. Median age at notification, in particular, offers a reproducible metric for monitoring the timeliness of detection and referral processes using routinely collected data.

## Conclusion

During the study period, the prevalence of CHD and CCHD in Argentina was 65.3 and 15.0 per 10,000 live births, respectively, with substantial variation across provinces. These differences are unlikely to reflect only epidemiological variation, and instead suggest differences in case detection, reporting and access to specialized care across provinces. Interpreting prevalence together with severity distribution and notification timing provides a more nuanced understanding of these patterns and supports the use of routine registry data to monitor the timeliness of detection and referral processes.
