# Supplementary material for: Provincial variability in congenital heart disease prevalence in Argentina, 2014–2019: A population-based analysis from national registry data
Source: PLOS Glob Public Health. 2026 Jun 22;6(6):e0005217. doi: 10.1371/journal.pgph.0005217 (PMC13286159; doi:10.1371/journal.pgph.0005217)
Supplement: Attachment — Submitted filename: REBUTTAL LETTER CHD.docx [file pgph.0005217.s002.docx]

**REBUTTAL LETTER TO MANUSCRIPT PGPH-D-25-02368**

Dear Editor and Reviewers,

We thank you for the thorough evaluation of our manuscript and for the insightful comments provided. We have carefully revised the manuscript in response to each point raised. Below, we provide a detailed, point-by-point response, indicating the changes made and where they can be found in the revised version.

**Comments from Reviewer #1:**

- *Study design and population (page 6): This does not appear to be an ecological study, as the unit of analysis is the neonate.*

We thank the reviewer for this observation. You are correct that the primary classification of our study design is cross-sectional. We have clarified this in the Methods section. Although the analysis was conducted using aggregated province-level indicators—an approach consistent with an ecological framework—we did not examine associations between these outcomes (CHD and CCHD prevalence) and specific exposures. For this reason, we now describe the study as a cross-sectional descriptive analysis based on province-level aggregated data, and have revised the text accordingly where appropriate. Importantly, this clarification does not alter the study methodology, the analytic procedures performed, or the results obtained.

- ***Age at diagnosis (page 7):*** *It is unclear whether reporting is immediate upon detection. Could there be a bias due to delayed case notification?*

We appreciate the reviewer’s insightful comment. We have clarified in the manuscript that “age at diagnosis” was extracted from the RNCC based on the date of case notification. We acknowledge that case notification may not always occur immediately after clinical detection, which could introduce bias in the estimated age at diagnosis. This limitation has been explicitly addressed in the Methods section.

- **Methodological considerations (page 8):**

1) *Data quality control: Is it possible that there were missing data in some of the variables (e.g., age at diagnosis, residence, or others)? If so, how was this handled?*

The data were obtained from the National Registry of Congenital Heart Disease (RNCC), which includes mandatory fields for each case entry. These mandatory variables comprise date of birth, province of residence and diagnosis, among others. The date and time of notification are automatically generated by the system. While other optional variables exist, only mandatory fields were included in this analysis to ensure completeness and consistency of the dataset. Therefore, there were no missing data for the variables analyzed in this study.

2) *Limitations: Evaluating the impact of the Voluntary Termination of Pregnancy law is not the objective proposed in this article, so it is not a “limitation” for the stated objective. In any case, it is important to mention it because the figures may not reflect the current situation, but rather the reality prior to the law, and in such a case, the presented figures would not be suitable for current decision-making.*

We appreciate the reviewer’s observation regarding the reference to the Voluntary Termination of Pregnancy Law. We agree that assessing its impact was not an objective of this study. Following the suggestion, we removed this point from the *Limitations* section and clarified it in the *Discussion*, emphasizing that the data correspond to a period prior to the implementation of the law. This contextual note highlights that the figures may differ from the current situation, given potential changes in prenatal diagnosis and pregnancy outcomes after legalization.

- **Results:**

1) *Table 1 (page 9): The table headers are in Spanish and should be written in English.*

We thank the reviewer for this observation. All table headers have been translated into English to ensure consistency with the rest of the manuscript.

2) *“Interquartile range [IQR]”: The figures presented appear to correspond to the first and third quartiles, rather than the interquartile range. If that is the case, modify the label to indicate Q1–Q3 instead of IQR.*

We thank the reviewer for this helpful observation. You are correct –the figures presented correspond to the first and third quartiles. We have revised the tables and text accordingly, replacing “IQR” with “Q1–Q3” to accurately reflect the data presented.

3) *Suggestion: To improve the visualization of the presented figures, include a map to show whether prevalences are randomly distributed across Argentina or if there are regions with more correlated results—for example, neighboring provinces with low (or high) neonatal heart disease prevalence.*

Thank you for this valuable suggestion. We have now included a map illustrating the spatial distribution of CHD prevalence across Argentine provinces. The map (new Figure 1) shows that prevalences follow a heterogeneous geographic pattern. The highest values are concentrated in the southernmost provinces (Tierra del Fuego and Chubut), while additional elevated prevalences appear in several northern and central provinces (e.g., Corrientes, Formosa, Chaco, La Rioja, La Pampa). In contrast, lower prevalences are observed in provinces such as Buenos Aires, Santa Fe, Jujuy, and the Buenos Aires metropolitan area. We also added an inset to improve visualization of CABA.

- **Discussion:**

1) *Page 13: “…it is essential to evaluate whether lower prevalence in these provinces relates to higher neonatal mortality.” This comment is very important, as it recognizes the importance of linking early detection of congenital heart diseases in neonates with neonatal morbidity and mortality. However, this was not addressed in the study—was this due to lack of data? Since its importance is acknowledged, it would be relevant to elaborate on why this association was not tested in this article, or alternatively, to test it.*

We appreciate the reviewer’s insightful comment. We agree that examining the relationship between CHD/CCHD prevalence and neonatal mortality would provide important context regarding early detection and outcomes. Although the national reporting system (SISA) registers whether a newborn survives the neonatal period, this variable is not included in the analytical dataset we accessed for the present study. As a result, neonatal mortality was not available as an outcome in our database, and individual-level linkage between CHD cases and neonatal survival could not be performed.

Additionally, provincial neonatal mortality statistics do not specify deaths attributable to congenital heart disease. Therefore, comparing CHD prevalence with overall neonatal mortality at the population level could lead to misleading inferences, and may not reflect access to CHD detection or treatment.

For these reasons, we limited our analysis to prevalence and explicitly acknowledged the potential relevance of this association in the Discussion. We have now clarified this point in the manuscript.

2) *Page 16: the importance of this association is also highlighted: “Targeted strategies prioritizing equity…can improve early diagnosis and access to specialized care, reducing preventable morbidity and mortality.” This statement should either be referenced or tested here.*

We thank the reviewer for this comment. We incorporated appropriate references supporting this statement. In particular, we incorporated the 2025 national consensus from the Argentine Society of Pediatrics, which states that early detection improves prognosis by enabling timely stabilization and neonatal management, preventing disease progression and death. We also included recent international evidence (Patel 2024) showing that prenatal diagnosis and coordinated perinatal planning prevent postnatal clinical deterioration in newborns with critical congenital heart disease. The Discussion section has been updated accordingly.

**Comments from Reviewer #2:**

***Weakness and areas needed for further elaboration:***

- **Introduction***: the authors mention that congenital cardiac abnormalities account for over 10% of deaths in the same age group (Intro, 2nd paragraph).
  Given that the authors stats (when calculated) in Argentina reveal 0.75/500 critical congenital abnormalities detected versus the world (1/500) - an elaboration of what causes this would be important. For instance, is this accounting for: genetic differences? environmental or socioeconomic differences? infectious pathology differences?*

We thank the reviewer for this thoughtful comment. The lower proportion of CCHD detection we report compared with international estimates likely reflects multiple factors. However, we cannot attribute the difference to a single cause based on the available data. Instead, several plausible explanations may contribute, including: (a) underdiagnosis or delayed diagnosis in settings with limited access to pediatric cardiology and echocardiography; (b) incomplete or heterogeneous reporting across jurisdictions; (c) differences in screening practices, as pulse oximetry screening is nationally recommended but only mandatory in some provinces, resulting in heterogeneous implementation across jurisdictions; and (d) potential variations in survival to the point of notification. At present, there is no evidence suggesting population-level genetic or infectious differences that would explain a lower prevalence of CCHD in Argentina. We have added a paragraph in the Discussion acknowledging these potential factors and emphasizing the need for improved detection systems and enhanced surveillance to better understand these differences.

- *The study mentions "barriers including limited infrastructure and human resources within the public health system to meet the national demand for diagnosis and treatment" (Intro- paragraph 4). However the database takes into consideration only Argentine Nationals and not foreigners (Materials and Methods - paragraph 1). Therefore, as the objective is states as "to estimate the prevalence of congenital heart defects and critical congenital heart defects among live births in the public health sector" (intro - last paragraph) - by not accounting for non-argentine patients attended to in the public health sector (especially by province), this has the potential to severely misrepresent the "burden" on the public health system. This question needs to be addressed (either by incorporating this data or recognizing it as a significant limitation) in order for the manuscript to carry relevance to recognizing the magnitude of public health utilization the pathology represents.*

We thank the reviewer for raising this point. Our prevalence estimates rely on live births recorded in the national vital statistics system (DEIS); therefore, infants born outside Argentina are not part of the denominator from which prevalence can be calculated, regardless of whether they later receive care in the public sector. Foreign-born infants diagnosed in Argentina were excluded to maintain internal consistency between numerator and denominator, ensure a homogeneous study population, and reduce selection bias that would arise from including children who are not part of the live-birth population from which prevalence is derived. This exclusion prevents overestimation and does not affect our prevalence estimates.

We agree that foreign-born patients may contribute to the clinical workload in certain jurisdictions. However, this relates to health-service utilization rather than prevalence estimation. We now acknowledge this distinction in the Discussion, noting that while foreign-born patients may influence burden of care locally, their exclusion does not alter the prevalence estimates derived from national birth data.

- *In order to strengthen their conclusion, a more thorough evaluation of neonatal screening disparities (for instance implementation of provincial health ministry policies) between provinces may help explain inter-province differences in detection, time to detection, and rates of overall and critical congenital abnormalities. An exploration of this data would significantly strengthen the value of this manuscript to the scientific literature. This may allow for further areas of investigation to focus resources on with respect to achieving a more equitable approach to countrywide population screening for said pathology.
  For insatance, in the conclusion, its mentioned that 27% of the pediatric cardiologists are in CABA - further exploration of cardiologist per Km2 or cardiologist per 1000 births would more clearly demonstrate this disparity.*

We thank the reviewer for this suggestion. Workforce figures were obtained from MAPA (Monitoreo para el Análisis y Planificación de Acciones), the national administrative registry of health professionals. Although MAPA provides useful descriptive information on the distribution of pediatric cardiologists, quantitative province-level analyses could be limited. The registry records matriculated professionals rather than active clinical practice, does not distinguish public versus private sector activity, and is affected by jurisdictional differences in registration processes. As a result, the number of registered cardiologists in a province does not reliably represent the diagnostic capacity relevant to CHD detection. Moreover, MAPA indicators are population-based and therefore not aligned with the live-birth denominators required for CHD epidemiology. For these methodological reasons, MAPA cannot be converted into indicators such as specialists per km² or per number of live births without introducing substantial bias.
Nevertheless, MAPA does illustrate marked interprovincial disparities –for example, CABA concentrates a disproportionately high share of pediatric cardiologists compared with most provinces. While these administrative data cannot be used for quantitative analysis, this qualitative pattern supports our interpretation that unequal specialist distribution might contribute to differences in diagnostic capacity. We have clarified this in the Discussion and now acknowledge specialist distribution as an important contextual factor and a priority for future investigation.

- *the study mentions that there is "at least one designated referral center wtih a cardiologist responsible for diagnosis and reporting" (Intro - last paragraph) - how does this relate to center per capita and center per geographic catchment? This data is important in understanding the magnitude of disparity in terms of reporting with respect to resources and healthcare accessibility.*

We thank the reviewer for this valuable comment. In Argentina, the organization of CHD care follows a regionalized model coordinated by the National CHD Program. During the study period, the network included 40 diagnostic centers distributed across all 24 jurisdictions with 15 surgical centers located in 9 jurisdictions. Many of these surgical centers are located in the most populous provinces (Ciudad Autónoma de Buenos Aires, Buenos Aires Province, Santa Fe, Córdoba, and Mendoza), reflecting the common organizational approach of concentrating high-complexity pediatric cardiac surgery in a limited number of specialized centers. Because these procedures require highly trained teams, advanced technology, and minimum procedural volumes to maintain quality, the distribution of surgical capacity does not follow a per-capita or per-kilometer criterion. As a result, the effective catchment areas of referral centers may vary across provinces, particularly those without local surgical services or with large geographic extension or low population density.

While our study was not designed to quantify catchment or accessibility, we have clarified these structural characteristics in the revised manuscript to better contextualize potential differences in early detection and reporting across provinces.

- *The study registers only cases reported in public sector facilities. is there accounting for studies recognized in the private sector ultimately treated in the public sector? (for instance diagnosed in Salta in a private clinic and then transferred to BsAs (Garrahan) for definitive treatment) are these included or excluded? If they are included - how does this impact the prevalence between provinces (especially in a country as centralized as Argentina?)*

We appreciate this important comment. During the study period (2014–2019), notification of congenital anomalies to the national surveillance system (SISA) was implemented primarily through public-sector facilities, which is why our database includes only cases from the public sector. The regulatory framework has since expanded with Law 27.713 (2023), which created federal registries and incorporated CHD detection into the mandatory benefits package, extending the program to public and private institutions. As this change occurred after the study period, our prevalence estimates necessarily reflect the public-sector births systematically reported during 2014–2019.

According to official data from the National Vital Statistics System (DEIS), a substantial proportion of live births in Argentina occur in the public sector, and these births constitute the denominators used for our provincial prevalence estimates. Children initially evaluated in the private sector may occasionally be transferred to public hospitals and therefore become eligible for notification. Conversely, some children born in the public sector may subsequently receive care in private facilities. These cross-subsystem movements occur in both directions and represent a small and non-systematic proportion of all CHD cases. Importantly, in the national registry cases are assigned to the patient’s usual place of residence, regardless of where diagnosis or treatment occurs. Therefore, a child born in one province but treated in a high-complexity center elsewhere continues to be classified under their province of residence.

For these reasons, any upward bias introduced by cross-subsystem notification is expected to be minimal and unlikely to materially affect provincial comparisons. We now clarify this point in the Discussion, characterizing our provincial estimates as a robust representation of the burden of CHD within the public sector.
