# Supplementary material for: Provincial variability in congenital heart disease prevalence in Argentina, 2014–2019: A population-based analysis from national registry data
Source: PLOS Glob Public Health. 2026 Jun 22;6(6):e0005217. doi: 10.1371/journal.pgph.0005217 (PMC13286159; doi:10.1371/journal.pgph.0005217)
Supplement: Attachment — Submitted filename: REBUTTAL LETTER CHD R1.docx [file pgph.0005217.s004.docx]

**REBUTTAL LETTER TO MANUSCRIPT PGPH-D-25-02368R1**

We thank the Editor for the careful evaluation of our revised manuscript and for the thoughtful suggestions aimed at strengthening its framing and global relevance. We have carefully revised the Discussion to address each point raised, with particular attention to regional contextualization, policy implications, and articulation of the study’s contribution to global congenital anomaly surveillance. All changes are reflected in the revised manuscript and highlighted in the tracked version.

1. **A more explicit regional (Latin American) framing, rather than primarily contrasting findings with European estimates**

We appreciate this helpful suggestion. We agree that the manuscript should more clearly situate Argentina’s findings within a regional Latin American context rather than primarily using European estimates as benchmarks. Accordingly, we revised the Discussion to explicitly frame Argentina within comparable middle-income and fragmented health systems, characterized by mixed public–private care, decentralized governance and heterogeneous surveillance models (hospital- vs population-based; active/passive/hybrid). We also incorporated reference to the Red Latinoamericana de Malformaciones Congénitas (ReLAMC) to contextualize how differences in coverage and case ascertainment influence reported prevalence across the region. European estimates are now presented only as contextual reference points rather than normative comparators.

These revisions appear in the Discussion (revised manuscript with track changes, Page 13, Lines 263–286; Page 16, Lines 338–347).

**2. Concrete examples of how the results could inform policy, planning, or surveillance strategies in comparable settings.**

We thank the Editor for this valuable recommendation. We have expanded the Discussion to include concrete examples of how registry-based data registry-based data can inform decision-making. Specifically, we added text describing:

(i) how provincial differences in prevalence and age at notification can guide phased implementation and monitoring of neonatal pulse oximetry screening programs;

(ii) how Argentina’s National Congenital Heart Disease Program illustrates the organization of referral networks linking provincial diagnostic centers with a limited number of high-complexity surgical centers;

(iii) how registry indicators can support targeted workforce distribution and resource allocation; and

(iv) how delayed age at notification can be operationalized as a pragmatic health-system performance indicator to monitor timeliness of detection across jurisdictions and over time.

These additions appear in the Discussion (Page 18, Lines 391–395; Page 21, Lines 459–480).

3. **Clear articulation of what this study contributes to global public health knowledge beyond documenting subnational variability.**

*We appreciate this suggestion and agree that the broader global public health implications of our findings should be more explicitly articulated. We have therefore revised the final section of the Discussion to clarify how reliance on public-sector registries within segmented health systems may lead to incomplete case ascertainment, potentially contributing to underestimation of CHD prevalence in global burden assessments.*

*We also emphasize the value of subnational analyses for identifying inequities in detection and access to specialized care. Finally, we highlight how integrating disease-specific notification systems with coordinated referral networks may strengthen congenital anomaly surveillance and support equity-oriented health planning in comparable middle-income settings.*

*These revisions are reflected in the final section of the Discussion (Page 22, Lines 491*–504)
