# Supplementary material for: Provincial variability in congenital heart disease prevalence in Argentina, 2014–2019: A population-based analysis from national registry data
Source: PLOS Glob Public Health. 2026 Jun 22;6(6):e0005217. doi: 10.1371/journal.pgph.0005217 (PMC13286159; doi:10.1371/journal.pgph.0005217)
Supplement: Attachment — Submitted filename: PGPH-D-25-02368 discussion guidance.docx [file pgph.0005217.s003.docx]

Please revise the Discussion according to the following structural guidance:

1. Begin with a clear statement of the study’s added value

The Discussion should open with a concise paragraph that clearly articulates:

- What this study uniquely contributes (relative to Groisman and Bakker).
- The importance of subnational (provincial) analysis.
- The use of median age at notification as a systems-level metric.

Currently, comparisons are presented, but the added value of this study is not sharply defined. The first paragraph should establish this contribution explicitly.

2. Consolidate and reorganize comparisons

The sections comparing this study with other studies and context should be integrated earlier with the paragraph beginning around line 281, so that international comparisons precede subnational interpretation. Comparisons should be concise and contained; they should not interrupt the analytical flow of the Discussion.

3. Center the discussion on interpretation of provincial heterogeneity

Rather than narrating province-by-province differences, reorganize the Discussion around system-level explanatory dimensions supported by your metrics. Specifically:

A. Detection and diagnostic capacity

Interpret prevalence variation (CHD vs CCHD) in relation to diagnostic infrastructure and prenatal detection capacity. Avoid simply restating results; instead, synthesize what these differences likely represent structurally.

B. Median age at notification as a system-level indicator

This should be a central analytical section. Clarify:

- What median notification age represents.
- Whether it approximates delayed diagnosis.
- How it relates to early mortality risk (if applicable).
- How it reflects implementation strength.

The pulse oximetry example is effective because it links policy, implementation, and measurable outcome. This model of interpretation should guide the rest of the Discussion.

The statement regarding 30% of newborns discharged undiagnosed and 50% mortality must be clearly connected to your study findings (e.g., whether notification delay plausibly reflects delayed detection). If no direct link can be made, this statistic should be contextualized more carefully.

C. Workforce distribution and referral networks

Variability in pediatric cardiologist availability, prenatal diagnostic access, and referral systems should form a coherent explanatory thread, not scattered speculation. Use the results to support this interpretation rather than repeating general statements about inequity.

4. Clarify registry structure and potential bias

Statements regarding cross-subsector notification bias must be reconciled. For example:

- If cross-subsector notification is cited as a possible explanation for CABA differences, this must be logically consistent with the later statement that such bias is unlikely to materially affect between-province comparisons.
- Consolidate all discussion of registry limitations into a single, concise section rather than revisiting them repeatedly.

5. Reduce redundancy and boilerplate policy language

The Discussion currently contains repeated references to inequities, strengthening surveillance, and infrastructure reinforcement without advancing the argument.

Please:

- Eliminate repetitive summaries of provincial variability.
- Remove generic statements (e.g., “Targeted strategies prioritizing equity…”).
- Focus on the specific metrics that proved informative (prevalence, CCHD proportion, median notification age).
- Articulate how these metrics can function as operational indicators of system performance.

The overall length of the Discussion should be reduced, prioritizing synthesis over repetition. I suggest doing a reverse outline exercise of your current discussion (<https://writing.wisc.edu/handbook/reverseoutlines/>) to better structure it and reduce redundancy. The revised Discussion should follow a clear progression:

1. What this study adds.
2. How it differs from prior Argentine and international work.
3. Interpretation of provincial heterogeneity (diagnostic capacity, notification timing, workforce/referral systems).
4. Registry structure and limitations (concise).
5. Focused, metric-based implications.
6. Short, strong synthesis paragraph.

Avoid oscillating between international comparisons, provincial contrasts, and registry caveats without clear structure.
