# Supplementary material for: Provincial variability in congenital heart disease prevalence in Argentina, 2014–2019: A population-based analysis from national registry data
Source: PLOS Glob Public Health. 2026 Jun 22;6(6):e0005217. doi: 10.1371/journal.pgph.0005217 (PMC13286159; doi:10.1371/journal.pgph.0005217)
Supplement: Attachment — Submitted filename: REBUTTAL LETTER TO MANUSCRIPT PGPH-D-25-02368R2.docx [file pgph.0005217.s005.docx]

**REBUTTAL LETTER TO MANUSCRIPT PGPH-D-25-02368R2**

Dear Editor,

Thank you for your careful and constructive feedback and, especially, for the time you took to provide such detailed guidance. We are very grateful for the clarity of your comments, which were extremely helpful in showing us how to improve the manuscript.

We have revised the Discussion section to address the remaining concerns, focusing on improving its structure, reducing repetition, and strengthening the central message. Your suggestions were key in helping us reorganize the section and make the overall argument more coherent and easier to follow.

In particular, we reorganized the Discussion to follow a clearer analytical progression, ensuring that comparisons, interpretation, limitations, and implications are presented in a structured and non-overlapping manner. We also reduced repetition and removed more general or policy-oriented statements that did not directly contribute to the interpretation of the findings.

We agree with the editor that the most informative analytical thread of the study lies in the integration of detection capacity, referral systems, and notification timing. Accordingly, we strengthened this framework throughout the Discussion, using median age at notification as a central system-level indicator and aligning other results with this perspective. The pulse oximetry example was retained and placed to more clearly illustrate the link between policy, implementation, and measurable outcomes.

Specifically, the Discussion was revised as follows:

- The opening paragraph was rewritten to clearly state the study’s added value.
- Comparisons with prior Argentine and international studies were consolidated and moved earlier in the section.
- The interpretation of provincial heterogeneity was reorganized to link observed variation in key metrics (prevalence, severity distribution, and notification timing) with underlying differences in diagnostic capacity and referral pathways.
- All discussion of registry structure and limitations was consolidated into a single, concise paragraph.
- The concluding paragraph was shortened and refocused to provide a clearer synthesis of the main findings.
- Minor revisions were also made throughout the manuscript to improve clarity and consistency, including updating reference numbering to reflect changes in citation order.

We believe these revisions have substantially improved the clarity, coherence, and focus of the Discussion, and we hope the manuscript is now suitable for publication.

Thank you again for your thoughtful guidance and for the opportunity to revise our work.

Kind regards,

María Clara Vita on behalf of all authors
