# Supplementary material for: Provincial variability in congenital heart disease prevalence in Argentina, 2014–2019: A population-based analysis from national registry data
Source: PLOS Glob Public Health. 2026 Jun 22;6(6):e0005217. doi: 10.1371/journal.pgph.0005217 (PMC13286159; doi:10.1371/journal.pgph.0005217)
Supplement: Attachment — Submitted filename: REBUTTAL LETTER TO MANUSCRIPT PGPH-D-25-02368R4.docx [file pgph.0005217.s006.docx]

**REBUTTAL LETTER TO MANUSCRIPT PGPH-D-25-02368R4**

Dear Editor,

Thank you for your careful review and helpful suggestions. We have revised the manuscript accordingly to improve clarity, consistency, and alignment between sections.

In particular, we revised the Introduction to better contextualize the study for an international audience and to more clearly connect the description of the national network with the study objectives.

We also revised the presentation of the results and tables to improve interpretability. Specifically, we clarified that age at notification is expressed in days and incorporated the median age ratio (CCHD/CHD) as an additional analytical indicator. This metric was also integrated into both the Results and Discussion to enhance the interpretation of differences in notification timing across provinces. In addition, we reordered the provinces in Tables 1 and 2 based on their respective key metrics (CCHD as percentage of CHD in Table 1 and median age ratio in Table 2), rather than alphabetical order, to facilitate interpretation for an international audience.

To address consistency between sections, we ensured that all findings discussed are first introduced in the Results section. Figure 2 was removed, and the Discussion was adjusted to maintain a clear distinction between results and interpretation.

We also revised terminology throughout the manuscript to improve clarity for an international readership, replacing “jurisdictions” with “provinces” in most instances and explicitly referring to the inclusion of the Autonomous City of Buenos Aires (CABA) where relevant.

We believe these revisions have further strengthened the manuscript and improved its clarity and coherence. We appreciate the opportunity to revise our work and hope that the manuscript is now suitable for publication.

Kind regards,

María Clara Vita on behalf of all authors
