# Supplementary material for: Provincial variability in congenital heart disease prevalence in Argentina, 2014–2019: A population-based analysis from national registry data
Source: PLOS Glob Public Health. 2026 Jun 22;6(6):e0005217. doi: 10.1371/journal.pgph.0005217 (PMC13286159; doi:10.1371/journal.pgph.0005217)
Supplement: Peer review history

# Peer review: Decision Letter 0

**DOI:** 10.1371/journal.pgph.0005217.r001

**Authors:** Maria Fernandez

27 Oct 2025

PGPH-D-25-02368

Provincial variability in congenital heart disease prevalence in Argentina, 2014–2019: A population-based analysis from national registry data

PLOS Global Public Health

Dear Dr. Vita,

Thank you for submitting your manuscript to PLOS Global Public Health. After careful consideration, we feel that it has merit but does not fully meet PLOS Global Public Health’s publication criteria as it currently stands. Therefore, we invite you to submit a revised version of the manuscript that addresses the points raised during the review process.

Please submit your revised manuscript by Dec 11 2025 11:59PM. If you will need more time than this to complete your revisions, please reply to this message or contact the journal office at globalpubhealth@plos.org. When you're ready to submit your revision, log on to https://www.editorialmanager.com/pgph/ and select the 'Submissions Needing Revision' folder to locate your manuscript file.

Please include the following items when submitting your revised manuscript:

- A rebuttal letter that responds to each point raised by the editor and reviewer(s). You should upload this letter as a separate file labeled 'Response to Reviewers'.
- A marked-up copy of your manuscript that highlights changes made to the original version. You should upload this as a separate file labeled 'Revised Manuscript with Track Changes'.
- An unmarked version of your revised paper without tracked changes. You should upload this as a separate file labeled 'Manuscript'.

Guidelines for resubmitting your figure files are available below the reviewer comments at the end of this letter.

We look forward to receiving your revised manuscript.

Kind regards,

Maria del Pilar Fernandez

Academic Editor

PLOS Global Public Health

Journal Requirements:

1. In the online submission form you indicate that your data is not available for proprietary reasons and have provided a contact point for accessing this data. Please note that your current contact point is a co-author on this manuscript. According to our Data Policy, the contact point must not be an author on the manuscript and must be an institutional contact, ideally not an individual. Please revise your data statement to a non-author institutional point of contact, such as a data access or ethics committee, and send this to us via return email. Please also include contact information for the third party organization, and please include the full citation of where the data can be found.

2. Please include a separate legend or caption for Figure 1 in your manuscript.

3. If the reviewer comments include a recommendation to cite specific previously published works, please review and evaluate these publications to determine whether they are relevant and should be cited. There is no requirement to cite these works unless the editor has indicated otherwise.

Additional Editor Comments (if provided):

[Note: HTML markup is below. Please do not edit.]

Reviewers' comments:

Reviewer's Responses to Questions

**Comments to the Author**

1. Does this manuscript meet PLOS Global Public Health’s publication criteria? Is the manuscript technically sound, and do the data support the conclusions? The manuscript must describe methodologically and ethically rigorous research with conclusions that are appropriately drawn based on the data presented.

Reviewer #1: Yes

Reviewer #2: Yes

**********

2. Has the statistical analysis been performed appropriately and rigorously?

Reviewer #1: Yes

Reviewer #2: Yes

**********

3. Have the authors made all data underlying the findings in their manuscript fully available (please refer to the Data Availability Statement at the start of the manuscript PDF file)?

The PLOS Data policy requires authors to make all data underlying the findings described in their manuscript fully available without restriction, with rare exception. The data should be provided as part of the manuscript or its supporting information, or deposited to a public repository. For example, in addition to summary statistics, the data points behind means, medians and variance measures should be available. If there are restrictions on publicly sharing data—e.g. participant privacy or use of data from a third party—those must be specified.

Reviewer #1: Yes

Reviewer #2: Yes

**********

4. Is the manuscript presented in an intelligible fashion and written in standard English?

PLOS Global Public Health does not copyedit accepted manuscripts, so the language in submitted articles must be clear, correct, and unambiguous. Any typographical or grammatical errors should be corrected at revision, so please note any specific errors here.

Reviewer #1: Yes

Reviewer #2: Yes

**********

5. Review Comments to the Author

Please use the space provided to explain your answers to the questions above. You may also include additional comments for the author, including concerns about dual publication, research ethics, or publication ethics. (Please upload your review as an attachment if it exceeds 20,000 characters)

Reviewer #1: The article presented by the authors is highly valuable for public policy makers in Argentina regarding the early detection of congenital heart diseases in neonates.

Some comments regarding the work:

Study design and population (page 6): This does not appear to be an ecological study, as the unit of analysis is the neonate.

Variables – Age at diagnosis (page 7): It is unclear whether reporting is immediate upon detection. Could there be a bias due to delayed case notification?

Methodological considerations (page 8):

1) Data quality control: Is it possible that there were missing data in some of the variables (e.g., age at diagnosis, residence, or others)? If so, how was this handled?

2) Limitations: Evaluating the impact of the Voluntary Termination of Pregnancy law is not the objective proposed in this article, so it is not a “limitation” for the stated objective. In any case, it is important to mention it because the figures may not reflect the current situation, but rather the reality prior to the law, and in such a case, the presented figures would not be suitable for current decision-making.

Results:

1) Table 1 (page 9): The table headers are in Spanish and should be written in English

2) “Interquartile range [IQR]”: The figures presented appear to correspond to the first and third quartiles, rather than the interquartile range. If that is the case, modify the label to indicate Q1–Q3 instead of IQR.

3) Suggestion: To improve the visualization of the presented figures, include a map to show whether prevalences are randomly distributed across Argentina or if there are regions with more correlated results—for example, neighboring provinces with low (or high) neonatal heart disease prevalence.

Discussion: Page 13: “…it is essential to evaluate whether lower prevalence in these provinces relates to higher neonatal mortality.” This comment is very important, as it recognizes the importance of linking early detection of congenital heart diseases in neonates with neonatal morbidity and mortality. However, this was not addressed in the study—was this due to lack of data? Since its importance is acknowledged, it would be relevant to elaborate on why this association was not tested in this article, or alternatively, to test it.

On page 16, the importance of this association is also highlighted: “Targeted strategies prioritizing equity…can improve early diagnosis and access to specialized care, reducing preventable morbidity and mortality.” This statement should either be referenced or tested here.

Reviewer #2: This manuscript evaluates the database from the Argentine national registry of congenital heart disease.

As this is a database that is not publicly available, the data is original and reports important information and insights from said database.

Strengths:

- The conclusions drawn from said manuscript are important in identifying and recognizing some of the disparities in diagnostics and access to treatment of congenital disease in Argentina. For instance, there is importance in highlighting the differences in time to diagnosis of all versus severe congenital cardiac abnormalities.

- Highlighting the need to strengthen early detection and thereby access to appropriate further diagnostics and eventual management of said congenital cardiac abnormalities is a valid and important addition to the scientific literature.

Weakness and areas needed for further elaboration:

- in their introduction, the authors mention that congenital cardiac abnormalities account for over 10% of deaths in the same age group (Intro, 2nd paragraph).

- Given that the authors stats (when calculated) in Argentina reveal 0.75/500 critical congenital abnormalities detected versus the world (1/500) - an elaboration of what causes this would be important. For instance, is this accounting for: genetic differences? environmental or socioeconomic differences? infectious pathology differences?

- the study mentions "barriers including limited infrastructure and human resources within the public health system to meet the national demand for diagnosis and treatment" (Intro- paragraph 4). However the database takes into consideration only Argentine Nationals and not foreigners (Materials and Methods - paragraph 1). Therefore, as the objective is states as "to estimate the prevalence of congenital heart defects and critical congenital heart defects among live births in the public health sector" (intro - last paragraph) - by not accounting for non-argentine patients attended to in the public health sector (especially by province), this has the potential to severely misrepresent the "burden" on the public health system. This question needs to be addressed (either by incorporating this data or recognizing it as a significant limitation) in order for the manuscript to carry relevance to recognizing the magnitude of public health utilization the pathology represents.

- in order to strengthen their conclusion, a more thorough evaluation of neonatal screening disparities (for instance implementation of provincial health ministry policies) between provinces may help explain inter-province differences in detection, time to detection, and rates of overall and critical congenital abnormalities. An exploration of this data would significantly strengthen the value of this manuscript to the scientific literature. This may allow for further areas of investigation to focus resources on with respect to achieving a more equitable approach to countrywide population screening for said pathology.

For insatance, in the conclusion, its mentioned that 27% of the pediatric cardiologists are in CABA - further exploration of cardiologist per Km2 or cardiologist per 1000 births would more clearly demonstrate this disparity.

- the study mentions that there is "at least one designated referral center wtih a cardiologist responsible for diagnosis and reporting" (Intro - last paragraph) - how does this relate to center per capita and center per geographic catchment? This data is important in understanding the magnitude of disparity in terms of reporting with respect to resources and healthcare accessibility.

- the study registers only cases reported in public sector facilities. is there accounting for studies recognized in the private sector ultimately treated in the public sector? (for instance diagnosed in Salta in a private clinic and then transferred to BsAs (Garrahan) for definitive treatment) are these included or excluded? If they are included - how does this impact the prevalence between provinces (especially in a country as centralized as Argentina?)

Overall, the work is important to scientific literature in that it gives descriptive context to the database in question. However, it does appear to fall short as an abbreviated report of said database, and in order to significantly contribute to the scientific literature - a more in-depth of analysis cross-referencing available public health and professional data (with a more comprehensive epidemiologic and sociologic approach) is necessary to give context to the limited conclusions and define the impact of congenital cardiomyopathies in the Argentine public health sector.

**********

6. PLOS authors have the option to publish the peer review history of their article (what does this mean?). If published, this will include your full peer review and any attached files.

**Do you want your identity to be public for this peer review?**  If you choose “no”, your identity will remain anonymous but your review may still be made public.

For information about this choice, including consent withdrawal, please see our Privacy Policy.

Reviewer #1: No

Reviewer #2: No

**********

[NOTE: If reviewer comments were submitted as an attachment file, they will be attached to this email and accessible via the submission site. Please log into your account, locate the manuscript record, and check for the action link "View Attachments". If this link does not appear, there are no attachment files.]

-->Figure Resubmissions:

While revising your submission, we strongly recommend that you use PLOS’s NAAS tool (https://ngplosjournals.pagemajik.ai/artanalysis) to test your figure files. NAAS can convert your figure files to the TIFF file type and meet basic requirements (such as print size, resolution), or provide you with a report on issues that do not meet our requirements and that NAAS cannot fix.

After uploading your figures to PLOS’s NAAS tool - https://ngplosjournals.pagemajik.ai/artanalysis, NAAS will process the files provided and display the results in the "Uploaded Files" section of the page as the processing is complete. If the uploaded figures meet our requirements (or NAAS is able to fix the files to meet our requirements), the figure will be marked as "fixed" above. If NAAS is unable to fix the files, a red "failed" label will appear above. When NAAS has confirmed that the figure files meet our requirements, please download the file via the download option, and include these NAAS processed figure files when submitting your revised manuscript.

# Author comment: Author response to Decision Letter 1

**DOI:** 10.1371/journal.pgph.0005217.r002

4 Dec 2025

Attachment Submitted filename: REBUTTAL LETTER CHD.docx

# Editor report: Decision Letter 1

**DOI:** 10.1371/journal.pgph.0005217.r003

**Authors:** Maria Fernandez

22 Jan 2026

PGPH-D-25-02368R1

Provincial variability in congenital heart disease prevalence in Argentina, 2014–2019: A population-based analysis from national registry data

PLOS Global Public Health

Dear Dr. Vita,

Thank you for submitting your manuscript to PLOS Global Public Health. After careful consideration, we feel that it has merit but does not fully meet PLOS Global Public Health’s publication criteria as it currently stands. Therefore, we invite you to submit a revised version of the manuscript that addresses the points raised during the review process.

Please submit your revised manuscript by Mar 08 2026 11:59PM. If you will need more time than this to complete your revisions, please reply to this message or contact the journal office at globalpubhealth@plos.org. When you're ready to submit your revision, log on to https://www.editorialmanager.com/pgph/ and select the 'Submissions Needing Revision' folder to locate your manuscript file.

Please include the following items when submitting your revised manuscript:

- A letter that responds to each point raised by the editor and reviewer(s). You should upload this letter as a separate file labeled 'Response to Reviewers'.
- A marked-up copy of your manuscript that highlights changes made to the original version. You should upload this as a separate file labeled 'Revised Manuscript with Track Changes'.
- An unmarked version of your revised paper without tracked changes. You should upload this as a separate file labeled 'Manuscript'.

Guidelines for resubmitting your figure files are available below the reviewer comments at the end of this letter.

We look forward to receiving your revised manuscript.

Kind regards,

Maria del Pilar Fernandez

Academic Editor

PLOS Global Public Health

Journal Requirements:

If the reviewer comments include a recommendation to cite specific previously published works, please review and evaluate these publications to determine whether they are relevant and should be cited. There is no requirement to cite these works unless the editor has indicated otherwise.

Additional Editor Comments (if provided):

Thank you for submitting the revised version of your manuscript. The revision demonstrates that the authors have responded carefully and constructively to the reviewers’ technical comments. In particular, the clarification of methods, the expanded discussion of surveillance limitations, and the additional contextualization of provincial heterogeneity improve the clarity, transparency, and internal coherence of the study.

The manuscript presents a robust descriptive analysis of congenital heart disease and critical congenital heart disease using national registry data, and the epidemiological findings are clearly presented and well supported by the data. However, from an editorial perspective, the revised manuscript would still benefit from further strengthening its framing and synthesis to clearly demonstrate its relevance beyond the national context, in line with the scope and expectations of PLOS Global Public Health. While the Discussion has been expanded, it remains largely focused on explaining observed patterns within Argentina. The broader contribution to regional and global public health, particularly how these findings inform congenital anomaly surveillance, early detection strategies, and health system performance in comparable middle-income or decentralized health systems, is not yet sufficiently articulated.

In particular, the manuscript would be strengthened by:

1. A more explicit regional (Latin American) framing, rather than primarily contrasting findings with European estimates:

The current framing risks reinforcing a high-income country benchmark without adequately engaging with comparable middle-income or fragmented health systems. The authors should explicitly discuss how Argentina’s experience parallels or diverges from other Latin American countries with mixed public–private health systems, centralized versus decentralized congenital anomaly surveillance, uneven distribution of pediatric subspecialty care, etc.

2. Concrete examples of how the results could inform policy, planning, or surveillance strategies in comparable settings (rather than general statements):

For instance:

- How could registry data like these be used to guide phased expansion of neonatal pulse oximetry screening, targeted workforce deployment, or regionalization of surgical services?

- What lessons does Argentina’s National Congenital Heart Disease Program offer for other countries attempting to build referral networks with limited high-complexity centers?

- How might delayed age at notification be operationalized as a health system performance indicator relevant to global maternal–child health monitoring?

You don't need to address these questions, these are examples to help frame this topic more broadly.

3. Clear articulation of what this study contributes to global public health knowledge beyond documenting subnational variability: The manuscript should more explicitly articulate its contribution to global congenital anomaly surveillance, including the strengths and limitations of public-sector registries in segmented health systems, the implications for potential underestimation in global CHD prevalence estimates, and the added value of subnational analyses for equity-oriented public health planning.

Addressing these points would move the manuscript beyond a high-quality national description toward a contribution with clearer relevance and transferability for a global public health audience, providing insights into congenital anomaly surveillance, early diagnosis, and health system organization in middle-income countries. Without this broader regional and global framing, the manuscript risks being perceived as a high-quality national report rather than a contribution to global public health scholarship. We encourage the authors to focus on these aspects in a further revision.

[Note: HTML markup is below. Please do not edit.]

Reviewers' comments:

[NOTE: If reviewer comments were submitted as an attachment file, they will be attached to this email and accessible via the submission site. Please log into your account, locate the manuscript record, and check for the action link "View Attachments". If this link does not appear, there are no attachment files.]

-->Figure Resubmissions:

While revising your submission, we strongly recommend that you use PLOS’s NAAS tool (https://ngplosjournals.pagemajik.ai/artanalysis) to test your figure files. NAAS can convert your figure files to the TIFF file type and meet basic requirements (such as print size, resolution), or provide you with a report on issues that do not meet our requirements and that NAAS cannot fix.

After uploading your figures to PLOS’s NAAS tool - https://ngplosjournals.pagemajik.ai/artanalysis, NAAS will process the files provided and display the results in the "Uploaded Files" section of the page as the processing is complete. If the uploaded figures meet our requirements (or NAAS is able to fix the files to meet our requirements), the figure will be marked as "fixed" above. If NAAS is unable to fix the files, a red "failed" label will appear above. When NAAS has confirmed that the figure files meet our requirements, please download the file via the download option, and include these NAAS processed figure files when submitting your revised manuscript.

# Author comment: Author response to Decision Letter 2

**DOI:** 10.1371/journal.pgph.0005217.r004

11 Feb 2026

Attachment Submitted filename: REBUTTAL LETTER CHD R1.docx

# Editor report: Decision Letter 2

**DOI:** 10.1371/journal.pgph.0005217.r005

**Authors:** Maria Fernandez

19 Feb 2026

PGPH-D-25-02368R2

Provincial variability in congenital heart disease prevalence in Argentina, 2014–2019: A population-based analysis from national registry data

PLOS Global Public Health

Dear Dr. Vita,

Thank you for submitting your manuscript to PLOS Global Public Health. After careful consideration, we feel that it has merit but does not fully meet PLOS Global Public Health’s publication criteria as it currently stands. Therefore, we invite you to submit a revised version of the manuscript that addresses the points raised during the review process.

==============================

EDITOR: Please insert comments here and delete this placeholder text when finished. Be sure to:

- Indicate which changes you require for acceptance versus which changes you recommend
- Address any conflicts between the reviews so that it's clear which advice the authors should follow
- Provide specific feedback from your evaluation of the manuscript

Please ensure that your decision is justified on PLOS Global Public Health’s publication criteria and not, for example, on novelty or perceived impact.

==============================

Please submit your revised manuscript by Apr 05 2026 11:59PM. If you will need more time than this to complete your revisions, please reply to this message or contact the journal office at globalpubhealth@plos.org. When you're ready to submit your revision, log on to https://www.editorialmanager.com/pgph/ and select the 'Submissions Needing Revision' folder to locate your manuscript file.

Please include the following items when submitting your revised manuscript:

- A letter that responds to each point raised by the editor and reviewer(s). You should upload this letter as a separate file labeled 'Response to Reviewers'.
- A marked-up copy of your manuscript that highlights changes made to the original version. You should upload this as a separate file labeled 'Revised Manuscript with Track Changes'.
- An unmarked version of your revised paper without tracked changes. You should upload this as a separate file labeled 'Manuscript'.

Guidelines for resubmitting your figure files are available below the reviewer comments at the end of this letter.

We look forward to receiving your revised manuscript.

Kind regards,

Maria del Pilar Fernandez

Academic Editor

PLOS Global Public Health

Journal Requirements:

If the reviewer comments include a recommendation to cite specific previously published works, please review and evaluate these publications to determine whether they are relevant and should be cited. There is no requirement to cite these works unless the editor has indicated otherwise.

Additional Editor Comments (if provided):

Thank you for the revised manuscript. The authors have clearly incorporated the requested additions, particularly with respect to regional framing and articulation of broader relevance. The manuscript is substantially improved. However, the Discussion section still requires further revision before the manuscript can be accepted for publication. While many relevant elements are now present, the section remains overly long, repetitive, and insufficiently focused. As currently structured, the central analytical message becomes difficult to follow.

Several issues contribute to this:

• The Discussion moves back and forth between international comparisons, provincial differences, registry limitations, and policy implications without a clear hierarchy of argument.

• Results are sometimes restated rather than synthesized.

• Conclusions repeatedly return to general statements about inequities in access or surveillance capacity, without sharpening what the study’s specific metrics actually demonstrate.

In some cases, what appear to be contradictions (for example, regarding cross-subsector notification bias in CABA) likely stem from this lack of structural focus rather than true analytical inconsistency. The narrative would benefit from tighter organization so that explanations are cumulative and clearly anchored in the study’s data.

Importantly, the strongest analytical thread in the manuscript (linking variability in specialist distribution, diagnostic capacity, pulse oximetry implementation, and median age at notification) should serve as the backbone of the Discussion. The pulse oximetry example is particularly effective because it connects policy, implementation, and measurable outcomes. Similar clarity and integration should guide the rest of the section.

In order to be accepted, the Discussion should be substantially streamlined. Boilerplate policy language and repetitive summaries of provincial variability should be reduced in favor of a concise synthesis that:

• Clearly states what this study uniquely adds (relative to prior Argentine and international analyses),

• Focuses on the most informative metrics (e.g., notification timing as a systems-level indicator),

• And articulates the implications in a structured and logically progressive manner.

Overall, the manuscript is close to being publishable, but the Discussion must be more focused, more concise, and more analytically coherent before it can be accepted. Please see the attached document for more detailed guidance.

[Note: HTML markup is below. Please do not edit.]

Reviewers' comments:

[NOTE: If reviewer comments were submitted as an attachment file, they will be attached to this email and accessible via the submission site. Please log into your account, locate the manuscript record, and check for the action link "View Attachments". If this link does not appear, there are no attachment files.]

-->Figure Resubmissions:

While revising your submission, we strongly recommend that you use PLOS’s NAAS tool (https://ngplosjournals.pagemajik.ai/artanalysis) to test your figure files. NAAS can convert your figure files to the TIFF file type and meet basic requirements (such as print size, resolution), or provide you with a report on issues that do not meet our requirements and that NAAS cannot fix.

After uploading your figures to PLOS’s NAAS tool - https://ngplosjournals.pagemajik.ai/artanalysis, NAAS will process the files provided and display the results in the "Uploaded Files" section of the page as the processing is complete. If the uploaded figures meet our requirements (or NAAS is able to fix the files to meet our requirements), the figure will be marked as "fixed" above. If NAAS is unable to fix the files, a red "failed" label will appear above. When NAAS has confirmed that the figure files meet our requirements, please download the file via the download option, and include these NAAS processed figure files when submitting your revised manuscript.

Attachment Submitted filename: PGPH-D-25-02368 discussion guidance.docx

# Author comment: Author response to Decision Letter 3

**DOI:** 10.1371/journal.pgph.0005217.r006

2 Apr 2026

Attachment Submitted filename: REBUTTAL LETTER TO MANUSCRIPT PGPH-D-25-02368R2.docx

# Editor report: Decision Letter 3

**DOI:** 10.1371/journal.pgph.0005217.r007

**Authors:** Maria Fernandez

6 Apr 2026

PGPH-D-25-02368R3

Provincial variability in congenital heart disease prevalence in Argentina, 2014–2019: A population-based analysis from national registry data

PLOS Global Public Health

Dear Dr. Vita,

Thank you for submitting your manuscript to PLOS Global Public Health. After careful consideration, we feel that it has merit but does not fully meet PLOS Global Public Health’s publication criteria as it currently stands. Therefore, we invite you to submit a revised version of the manuscript that addresses the points raised during the review process.

Please submit your revised manuscript by May 06 2026 11:59PM. If you will need more time than this to complete your revisions, please reply to this message or contact the journal office at globalpubhealth@plos.org. When you're ready to submit your revision, log on to https://www.editorialmanager.com/pgph/ and select the 'Submissions Needing Revision' folder to locate your manuscript file.

Please include the following items when submitting your revised manuscript:

- A letter that responds to each point raised by the editor and reviewer(s). You should upload this letter as a separate file labeled 'Response to Reviewers'.
- A marked-up copy of your manuscript that highlights changes made to the original version. You should upload this as a separate file labeled 'Revised Manuscript with Track Changes'.
- An unmarked version of your revised paper without tracked changes. You should upload this as a separate file labeled 'Manuscript'.

Guidelines for resubmitting your figure files are available below the reviewer comments at the end of this letter.

As the corresponding author, your ORCID iD is verified in the submission system and will appear in the published article. PLOS supports the use of ORCID, and we encourage all coauthors to register for an ORCID iD and use it as well. Please encourage your coauthors to verify their ORCID iD within the submission system before final acceptance, as unverified ORCID iDs will not appear in the published article. *Only*  the individual author can complete the verification step; PLOS staff *cannot*  verify ORCID iDs on behalf of authors.

We look forward to receiving your revised manuscript.

Kind regards,

Maria del Pilar Fernandez

Academic Editor

PLOS Global Public Health

**Journal Requirements:**

If the reviewer comments include a recommendation to cite specific previously published works, please review and evaluate these publications to determine whether they are relevant and should be cited. There is no requirement to cite these works unless the editor has indicated otherwise.

Please review your reference list to ensure that it is complete and correct. If you have cited papers that have been retracted, please include the rationale for doing so in the manuscript text, or remove these references and replace them with relevant current references. Any changes to the reference list should be mentioned in the rebuttal letter that accompanies your revised manuscript. If you need to cite a retracted article, indicate the article’s retracted status in the References list and also include a citation and full reference for the retraction notice.

**Additional Editor Comments (if provided):**

The authors have done a great job in improving the discussion. I have some minor revisions requests before it can be accepted.

INTRODUCTION

The last two paragraphs needs to be better connected to the discussion and provide some more information for the international audience to have a better understanding of the study:

For example: "To address these gaps, the National Congenital Heart Disease Program, implemented in 2008, established a coordinated referral network with designated provincial diagnostic centers and a smaller number of high-complexity surgical centers (7,8). During the study period, the network comprised 40 diagnostic centers across all provinces but only 15 surgical centers concentrated in nine jurisdictions, creating heterogeneous catchment areas and potentially influencing detection and treatment patterns." --> indicate that this network was implemented in Argentina (it's implicit but not obvious). Also indicate that it's 40 diagnostic centers in all 23 provinces (not everyone knows how many provinces there are so this signals more than one diagnostic center per province), and when you indicate nine jurisdictions - do you mean provinces? Details matter...

The last paragraph when the objective is indicated, could be merged with the previous paragraph by indicating that this paper analyzes the performance across the network by "estimating the prevalence of congenital heart defects and critical congenital heart defects among live births in the public health sector from 2014 to 2019, and to analyzing differences in their distribution across Argentine provinces". It's a better way to connect with the later discussion too, you need to give the reader a better teaser for the study that can circle back to in the discussion.

TABLES

The tables need to be self-explanatory and not depend on the text for interpretation so I have a couple of suggestions:

Table 1: add units for the prevalence in the legend of the table (per 10,000 live births). You could also add the percent of CCHD of CHD cases to this table and remove fig 2. I would then organize the table not by alphabetical order but by prevalence of CHD OR the percent of CCHD over CHD (I would prefer the latter to match the discussion better).

Table 2: age implies years so again, in the legend or in the table indicate that it is age in days (for example "CHD – Median age (days, Q1–Q3)" or "CHD – Median age in days (Q1–Q3)"). In this table. I think it would also be interesting to add the ratio of the median age for CCHD to the median age to CHD as an indicator of how much faster the reporting occurs and how much it might be skewed towards more critical cases. For example, for Buenos Aires, it would be 39/93 days= 0.42 or (1-0.42)= 0.58... which means 58% younger (or faster diagnosis if you use it as an indicator). Again, if you decide to include this ratio (which will improve the quality of the manuscript and its conclusions), I would sort Table 2 by this metric instead of alphabetical order which means nothing to the international reader.

DISCUSSION

Some results are presented here without being first mentioned in the RESULTS. For example, Figure 2 is only referenced in the discussion but should be referenced in the results. We don't present new results in the discussion, if any figures or tables are referenced is just to emphasize the results but still needs to be introduced first (see my previous comment for Table 1). Another example is the number of pediatric cardiology specialists. This data could easily be included in Table 1 OR in Figure 1 (providing side by side geographic distributions of prevalence and workforce).

[Note: HTML markup is below. Please do not edit.]

**Reviewers' comments:**

[NOTE: If reviewer comments were submitted as an attachment file, they will be attached to this email and accessible via the submission site. Please log into your account, locate the manuscript record, and check for the action link "View Attachments". If this link does not appear, there are no attachment files.]

**Figure Resubmissions:**

While revising your submission, we strongly recommend that you use PLOS’s NAAS tool (https://ngplosjournals.pagemajik.ai/artanalysis) to test your figure files. NAAS can convert your figure files to the TIFF file type and meet basic requirements (such as print size, resolution), or provide you with a report on issues that do not meet our requirements and that NAAS cannot fix.

After uploading your figures to PLOS’s NAAS tool - https://ngplosjournals.pagemajik.ai/artanalysis, NAAS will process the files provided and display the results in the "Uploaded Files" section of the page as the processing is complete. If the uploaded figures meet our requirements (or NAAS is able to fix the files to meet our requirements), the figure will be marked as "fixed" above. If NAAS is unable to fix the files, a red "failed" label will appear above. When NAAS has confirmed that the figure files meet our requirements, please download the file via the download option, and include these NAAS processed figure files when submitting your revised manuscript.

# Author comment: Author response to Decision Letter 4

**DOI:** 10.1371/journal.pgph.0005217.r008

14 May 2026

Attachment Submitted filename: REBUTTAL LETTER TO MANUSCRIPT PGPH-D-25-02368R4.docx

# Editor report: Decision Letter 4

**DOI:** 10.1371/journal.pgph.0005217.r009

**Authors:** Maria Fernandez

21 May 2026

Provincial variability in congenital heart disease prevalence in Argentina, 2014–2019: A population-based analysis from national registry data

PGPH-D-25-02368R4

Dear Dr Vita,

We are pleased to inform you that your manuscript 'Provincial variability in congenital heart disease prevalence in Argentina, 2014–2019: A population-based analysis from national registry data' has been provisionally accepted for publication in PLOS Global Public Health.

Before your manuscript can be formally accepted you will need to complete some formatting changes, which you will receive in a follow up email. A member of our team will be in touch with a set of requests.

Please note that your manuscript will not be scheduled for publication until you have made the required changes, so a swift response is appreciated.

IMPORTANT: The editorial review process is now complete. PLOS will only permit corrections to spelling, formatting or significant scientific errors from this point onwards. Requests for major changes, or any which affect the scientific understanding of your work, will cause delays to the publication date of your manuscript.

If your institution or institutions have a press office, please notify them about your upcoming paper to help maximize its impact. If they'll be preparing press materials, please inform our press team as soon as possible -- no later than 48 hours after receiving the formal acceptance. Your manuscript will remain under strict press embargo until 2 pm Eastern Time on the date of publication. For more information, please contact globalpubhealth@plos.org.

Thank you again for supporting Open Access publishing; we are looking forward to publishing your work in PLOS Global Public Health.

Best regards,

Maria del Pilar Fernandez

Academic Editor

PLOS Global Public Health

***********************************************************

Reviewer Comments (if any, and for reference):
